# Prognostic Impact of Combinational Elastography in Patients with Heart Failure

**DOI:** 10.3390/jcm15020478

**Published:** 2026-01-07

**Authors:** Takahiro Sakamoto, Seita Yamasaki, Taiji Okada, Akihiro Endo, Hiroyuki Yoshitomi, Shuichi Sato, Kazuaki Tanabe

**Affiliations:** 1 Division of Cardiology, Shimane University Faculty of Medicine, 89-1, Enya-cho, Izumo 693-8501, Shimane, Japan; 2 Department of Internal Medicine, Izumo City General Medical Center, 613, Nadabuncho, Izumo 693-0003, Shimane, Japan

**Keywords:** heart failure, combinational elastography, liver stiffness

## Abstract

**Background:** Elastography is a non-invasive technique used to assess tissue stiffness. There are two main types of elastography: shear-wave elastography and strain imaging. Both are useful for evaluating the degree of liver fibrosis (LF). Shear-wave imaging is influenced by fibrosis and hepatic congestion, whereas strain imaging primarily reflects fibrosis progression and is less affected by congestion. We previously reported the clinical usefulness of combinational elastography in patients with heart failure (HF). However, its prognostic significance in this population remains unclear. Accordingly, in this prospective study, we aimed to evaluate the prognostic impact of combinational elastography in patients with HF. **Methods:** We included 77 patients with HF (median age: 79 years). Shear-wave imaging was used to obtain shear-wave velocity (Vs), whereas the liver fibrosis index (LF index) was derived from strain imaging. The Vs/LF index (V/L) was used as a prognostic indicator based on combinational elastography. Cardiac events were defined as cardiac death or hospitalization due to HF. **Results**: During a median follow-up of 716 days, 17 cardiac deaths or hospitalizations for HF were observed. The V/L demonstrated a cut-off value of 1.2 for predicting cardiac death or hospitalization for HF, with an area under the curve of 0.80, sensitivity of 0.82, and specificity of 0.68. Kaplan–Meier analysis demonstrated that patients with a high V/L (≥1.2) had significantly higher rates of hospitalization for HF than those with a low V/L (<1.2; log-rank test, *p* < 0.001). **Conclusions:** Combinational elastography demonstrated prognostic utility in patients with HF and may serve as a novel, non-invasive tool for assessing hepatic congestion.

## 1. Introduction

Heart failure (HF) is known to cause hepatic congestion and increased liver stiffness. Consequently, liver dysfunction is strongly associated with HF, and their coexistence is referred to as cardiohepatic syndrome [1,2]. The assessment of congestion remains challenging, as recently reviewed by Miller [3]. Recently, a non-invasive technique known as elastography has been used to assess liver stiffness. Elastography consists of two principal modalities, shear-wave elastography and strain imaging, both of which have been shown to be effective for assessing liver fibrosis (LF) [4]. Shear-wave imaging is affected not only by the degree of liver fibrosis but also by hepatic congestion, whereas strain imaging is relatively insensitive to congestion and mainly reflects the progression of fibrosis [5]. Accordingly, hepatic congestion can be assessed by combining shear-wave imaging with strain imaging, a technique known as combinational elastography [6]. We have previously reported the clinical usefulness of combinational elastography in patients with HF [7,8,9]. However, its prognostic impact on this population has not yet been investigated. Accordingly, we aimed to assess the prognostic impact of combinational elastography in patients with HF.

## 2. Materials and Methods

### 2.1. Patients and Protocol

This prospective study included 77 patients with HF who underwent combinational elastography, transthoracic echocardiography, and laboratory evaluations on the same day at the Masuda Red Cross Hospital between May 2017 and November 2018. HF was defined as a clinical syndrome characterized by typical signs and/or symptoms attributable to structural and/or functional cardiac abnormalities, supported by elevated natriuretic peptide levels and/or objective evidence of pulmonary or systemic congestion [10]. None of the participant had a history of or signs of liver disease, previous diagnosis of chronic liver disease, alcohol abuse (≥30 and ≥20 g/day for men and women, respectively), hepatic ultrasonography data indicating liver surface nodularity (a sign of severe fibrosis or ascites), anti-hepatitis C antibody positivity, or hepatitis B surface antigen reactivity. This exclusion was necessary to isolate the effect of congestion but limits generalizability to the broader HF population with comorbid liver disease. All patients undergoing combinational elastography were in a stable hemodynamic condition at the time of assessment and were subsequently followed for clinical outcomes. We evaluated the association between combinational elastography findings and the occurrence of cardiac death or hospitalization due to HF. Patients who received combinational elastography during periods of hemodynamic instability, including acute heart failure decompensation, were excluded.

The study protocol conformed to the principles outlined in the Declaration of Helsinki and was approved by the Ethics Committee of Masuda Red Cross Hospital (approval number: 49). Informed consent was obtained from all participants before their inclusion in the study from the analysis.

### 2.2. Combinational Elastography

**Figure 1:**
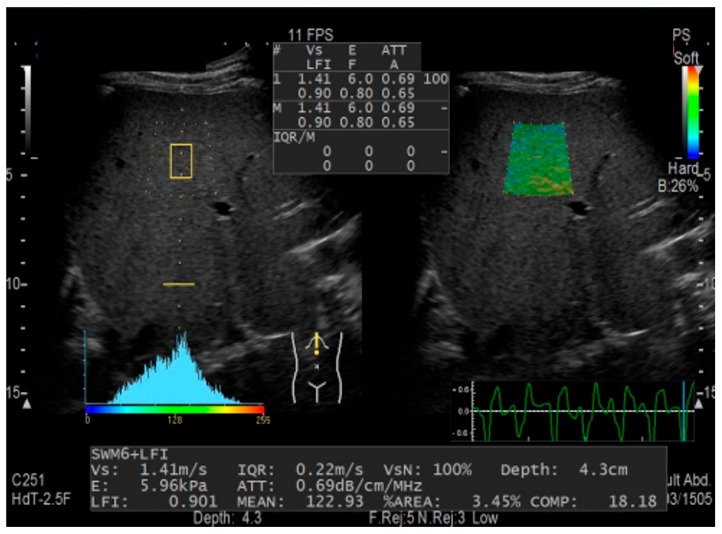
Liver stiffness is measured using combinational elastography. Shear-wave velocity is measured using shear-wave imaging. The liver fibrosis index is calculated using a multiple regression equation with nine feature values obtained from strain imaging.

The detailed methodology for combinational elastography was reported in our previous study [8]. All examinations were performed combinational elastography using an ARIETTA S70 or 850 ultrasonography system (Fujifilm Ltd., Tokyo, Japan) by a single experienced examiner (who had performed >500 assessments), blinded to all clinical data. This system is commercially available and testing costs can be reimbursed through insurance. Liver stiffness was assessed by shear-wave imaging through measurement of shear-wave velocity (Vs). Shear waves were generated from a focused ultrasound pulse once the measurement button was pressed, and the Vs value was displayed within approximately 2 s. The LF index was calculated using a multiple regression equation with nine feature values obtained by strain imaging used to diagnose LF in patients with chronic hepatitis C [11,12]. Combinational elastography was conducted via the right intercostal space, inferior to the right anterior axillary line, with the patient in a supine position and the right arm maximally abducted from the liver. A region of interest devoid of large blood vessels was located 1–2 cm below the liver surface (Figure 1). A successful recording of Vs was defined as ≥5 successful readings with a net effective shear-wave velocity of ≥50% [13]. The examiner selected 10 high-quality images to estimate the median LF index. Successful recording of Vs and 10 high-quality images were required to validate combinational elastography. Vs, and the LF index increased stepwise with increasing LF severity [4,13]. The Vs/LF index (V/L) is calculated as the ratio of Vs (which reflects hepatic congestion and fibrosis) to the LF index (which reflects fibrosis alone). This combined elastography measure was used as a prognostic indicator for HF.

### 2.3. Laboratory Tests, Echocardiography, and Composite Congestion Score

In addition to combinational elastography, laboratory data, echocardiographic findings, and the composite congestion score (CCS) were obtained on the same day. The CCS was calculated as described by Ambrosy et al., summing points for dyspnea, orthopnea, jugular venous pressure, hepatojugular reflux, peripheral edema, and rates [14]. Laboratory tests included liver function and brain natriuretic peptide level testing. All echocardiographic examinations were acquired by experienced sonographers in accordance with the guidelines of the American Society of Echocardiography/European Association of Cardiovascular Imaging [15]. The biplane method of discs was applied to apical four- and two-chamber images to determine left ventricular ejection fraction. Similarly, left atrial volume was measured using the biplane method of discs, and the left atrial volume was divided by the body surface area to calculate the left atrial volume index. Trans-mitral Doppler flow was used to obtain peak early and late diastolic velocities, while early diastolic mitral annular velocities were recorded at both the septal and lateral annuli. The left ventricular outflow tract velocity–time integral was determined by positioning the pulsed-wave Doppler sample volume just below the aortic valve within the outflow tract. In addition, inferior vena cava (IVC) diameter was assessed from the subcostal window, measured within 3 cm of the junction between the right atrium and IVC during quiet respiration.

### 2.4. Statistical Analysis

Continuous variables were expressed as medians (interquartile range) and compared using the Mann–Whitney U test. Categorical variables were expressed as numbers and percentages. Kaplan–Meier survival analysis was used to evaluate event-free survival, and differences between groups were assessed by the log-rank test. Receiver operating characteristic (ROC) curves were analyzed to assess the ability of the V/L ratio to predict the prognosis of cardiac death or hospitalization for HF, and the area under the ROC curve (AUC) was calculated. Given the limited number of events, multivariable Cox regression analysis was performed using a parsimonious model including clinically relevant variables, with particular focus on the V/L ratio and the CCS. Statistical analyses were conducted using EZR (version 1.54; Saitama Medical Center, Jichi Medical University, Saitama, Japan) and R software (version 4.0.3; R Foundation for Statistical Computing, Vienna, Austria), accessed through a graphical user interface [16]. Statistical significance was defined as a two-sided *p*-value < 0.05.

## 3. Results

### 3.1. Baseline Characteristics

The baseline characteristics of the participants in each cohort are shown in Table 1. Laboratory data, echocardiographic findings, and CCS data are summarized in Table 2. Patients with New York Heart Association class ≥ II had significantly higher V/L compared with those with New York Heart Association class I (1.4 [1.1–1.7] vs. 1.1 [0.9–1.4], *p* = 0.022).

### 3.2. Combinational Elastography and Clinical Outcomes

**Figure 2:**
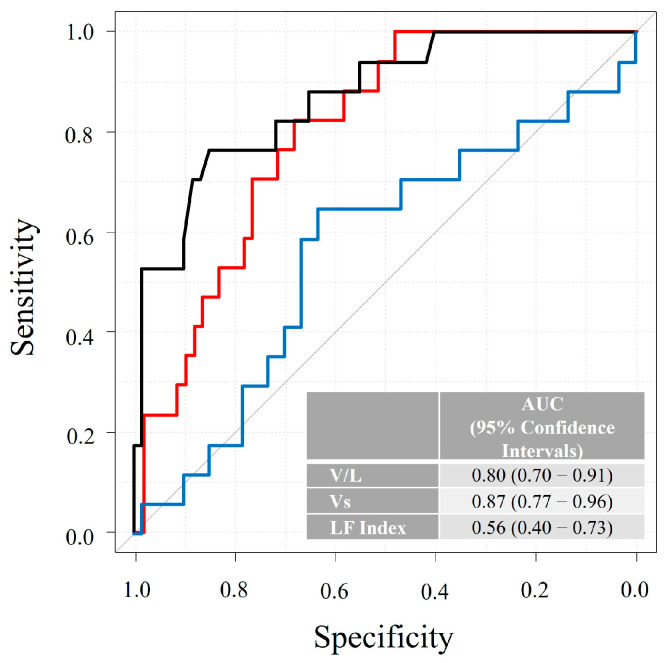
The diagnostic accuracy of the Shear-wave velocity (Vs)/liver fibrosis (LF) index (V/L) for predicting cardiac death or hospitalization for heart failure. The diagnostic accuracy of the V/L (red line), Vs value (black line) and LF index (blue line) for predicting cardiac death or hospitalizations for heart failure shows an area under the curve (AUC) of 0.80, 0.87 and 0.56 with a sensitivity of 0.82, 0.77 and 0.65, and a specificity of 0.68, 0.85 and 0.63, respectively.

**Figure 3:**
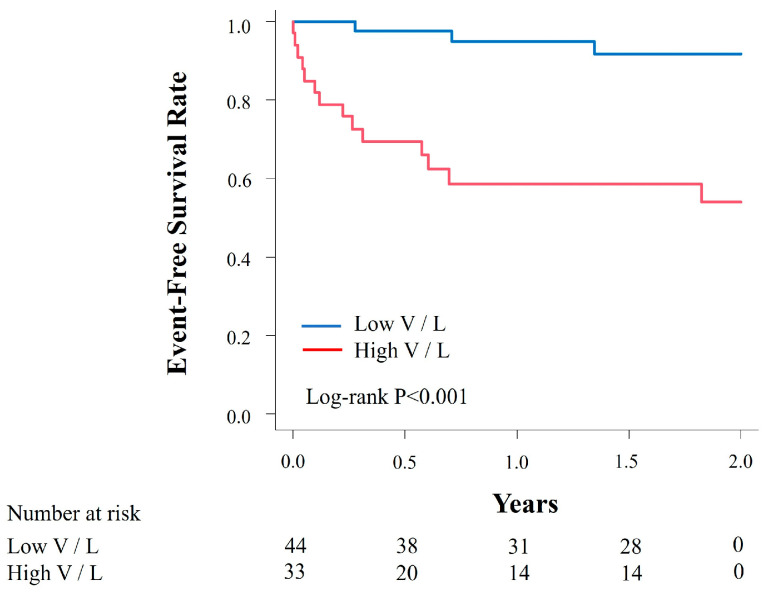
Kaplan–Meier plot of event-free (cardiac death or hospitalization for heart failure) survival in patients in the high Shear-wave velocity/liver fibrosis index (V/L) group (red line) and low V/L (blue line) group. Kaplan–Meier survival analysis demonstrated a significantly higher rate of cardiac death or heart failure–related hospitalization in patients with a high V/L ratio (≥1.2).

**Figure 4:**
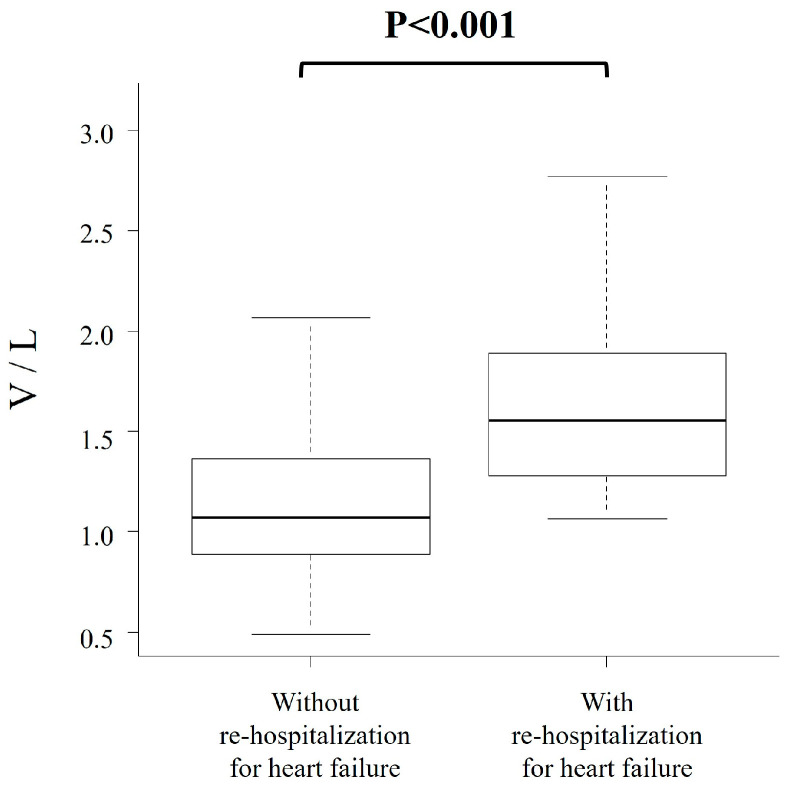
Patients re-hospitalized for heart failure show significantly higher Shear-wave velocity/liver fibrosis index (V/L) compared with those who are not re-hospitalized.

During a median follow-up of 716 days, 17 cardiac deaths or hospitalizations due to HF were observed, including 2 cardiac deaths and 15 hospitalizations due to HF. The diagnostic accuracy of V/L, Vs value and LF index for predicting cardiac death or hospitalization for HF showed an AUC of 0.80, 0.87 and 0.56 with a sensitivity of 0.82, 0.77 and 0.65, and a specificity of 0.68, 0.85 and 0.63, respectively (Figure 2). The AUC of the V/L was significantly higher than that of the LF index (*p* < 0.001), whereas no statistically significant difference was observed between the V/L and Vs alone (*p* = 0.11). The ROC curve analysis identified a cut-off value of 1.2 for V/L, 1.8 for Vs value and 1.3 for LF index, respectively. For comparison, the AUC for brain natrium peptide alone was 0.83 and for the CCS was 0.75 in predicting the primary endpoint. Kaplan–Meier analysis showed that patients in the high V/L group (≥1.2) had a significantly higher incidence of cardiac deaths or hospitalization for HF (*p* < 0.001, log-rank test) than those in the low V/L group (Figure 3). In the multivariable Cox proportional hazards analysis including both the V/L ratio and the CCS, both parameters remained independently associated with the primary outcome (V/L: *p* = 0.006; CCS: *p* < 0.001). Patients who were re-hospitalized for HF showed a significantly higher V/L than those who were not re-hospitalized (*p* < 0.001; Figure 4).

## 4. Discussion

To our knowledge, this is the first study to demonstrate the prognostic utility of combinational elastography (V/L ratio) in patients with HF. The V/L may serve as a complementary tool for congestion assessment, particularly in patients in whom conventional markers are inconclusive. Several studies have examined the association between hepatic elastography and patient outcomes. Liver stiffness measured using shear-wave imaging at admission or before discharge for HF—is associated with adverse events and long-term outcomes, respectively [17,18,19]. However, these studies did not include patients with an underlying liver disease. Combinational elastography can estimate hepatic congestion, even in patients with hepatic disorders such as alcoholic cirrhosis. Therefore, V/L, obtained through combinational elastography, may also offer prognostic value for such patients. Moreover, this technique may be beneficial in patients with chronic hepatic impairment secondary to persistently elevated right atrial pressure, such as after Fontan surgery. In this study, no statistically significant difference was observed between the V/L and Vs alone. Further studies including these populations are warranted.

In the present study, prognostic evaluation focused solely on hepatic congestion. However, previous studies have demonstrated that congestion in other organs—including the kidneys, lungs, gallbladder, spleen, and intestines—is also associated with prognosis in patients with HF [20,21,22,23,24,25]. It remains unclear which organ is the most significant determinant of prognosis. Further investigations are needed to evaluate congestion across multiple organs and to compare their diagnostic and prognostic accuracies. The assessment of multi-organ congestion via point-of-care ultrasound is an emerging paradigm in HF management, as highlighted in recent reviews [26]. Furthermore, although persistent hepatic congestion at discharge was associated with poor outcomes, we did not examine whether additional diuretic therapy to relieve congestion could improve prognosis. Notably, a previous study reported that patients with pulmonary congestion detected using remote dielectric sensing experienced better outcomes after intensification of diuretic therapy, and similar benefits may apply to patients with hepatic congestion [27]. The 2023 ESC Guidelines recommend relieving congestion as a central treatment goal but note that the optimal method to monitor congestion is not well established [28]. Our tool may help address this evidence gap.

### Limitations

Several limitations should be acknowledged. First, the relatively small sample size and limited number of clinical events may have affected the robustness of the analysis and introduced potential bias. Multicenter studies including more diverse patient populations are needed to validate the prognostic utility of the V/L. Although the small sample size reduced the robustness of the diagnostic accuracy analysis, the narrower confidence interval for V/L (0.70–0.91) shown in Figure 2 may be considered favorable. Second, the body mass index of the patients was all within the normal range. Fat thickness can affect the accuracy of the measurement for elastography. Excessive subcutaneous fat can affect the measurement results. Third, although Vs values have been reported to be generally comparable across different ultrasound devices, the LF index is a manufacturer-specific parameter and cannot be obtained using ultrasound systems from other vendors [29]. Therefore, V/L and its cut-off value identified in this study are applicable only to the ultrasound equipment used in the present investigation. When Vs is measured using different devices, the optimal cut-off value may differ. This represents an important limitation of the present study. Fourth, we had no data about right ventricular function therefore a relation with elastography may not be made. Fifth, our cohort represents a relatively low-risk population, with preserved IVC diameter and no significant pulmonary hypertension. It would be interesting to include patients with more severe disease and evaluate whether dynamic changes due to diuretic therapy may lead to improvement of V/L and its relation with prognosis. Finally, we did not perform head-to-head comparisons of the prognostic value of V/L against established biomarkers like brain natrium peptide or clinical scores like the CCS.

## 5. Conclusions

In conclusion, the combinational elastography-derived V/L ratio demonstrates promising prognostic utility in HF patients without primary liver disease. It may serve as a novel, non-invasive tool to quantify hepatic congestion. Future multi-center studies should validate these findings, compare V/L to standard prognostic markers, and explore its utility in guiding decongestive therapy.

## Figures and Tables

**Table 1:** Baseline characteristics of the participants.

	*n* = 77
Age, years	79 (73–86)
Male, *n* (%)	41 (52)
Body mass index, kg/m^2^	21 (19–24)
Systolic blood pressure, mmHg	113 (99–131)
Heart rate, bpm	70 (64–82)
New York Heart Association class I/II/III/IV	50/22/3/2
Medical history, *n* (%)	
Hypertension	63 (82)
Dyslipidemia	34 (44)
Diabetes mellitus	16 (21)
Atrial fibrillation	31 (40)
Chronic kidney disease	42 (55)
HF etiology, *n* (%)	
Cardiomyopathy	27 (35)
Valvular heart disease	15 (19)
Ischemia	13 (17)
Others	22 (29)
Medications	
ACEI or ARB, *n* (%)	45 (58)
β-blocker, *n* (%)	41 (53)
Mineral corticoid-receptor antagonists, *n* (%)	31 (40)
Loop diuretics, *n* (%)	54 (70)

Data are expressed as the median (interquartile range) or *n* (%). HF, heart failure; ACEI, angiotensin-converting enzyme inhibitor; ARB, angiotensin receptor blocker.

**Table 2:** Laboratory data, echocardiography findings, and composite congestion score.

	*n* = 77
Laboratory data	
Hemoglobin, g/dL	12 (10–13)
Platelet, ×10^3^/μL	20 (16–24)
Aspartate aminotransferase, U/L	23 (19–31)
Alanine aminotransferase, U/L	20 (13–29)
Gamma-glutamyl transpeptidase, U/L	33 (19–58)
Estimated glomerular filtration rate, mL/min/1.73 m^2^	45 (31–68)
Brain natriuretic peptide, pg/mL	308 (108–543)
Echocardiography	
Left ventricular end-diastolic volume, mL	75 (59–117)
Left ventricular end-systolic volume, mL	36 (23–68)
Left ventricular ejection fraction, %	49 (36–64)
Left atrial volume index, mL/m^2^	46 (34–61)
E/A	0.8 (0.6–1.6)
E/e’	13 (10–17)
Left ventricular outflow tract velocity-time integral, cm	16 (12–20)
Tricuspid regurgitation peak gradient, mmHg	26 (20–35)
Maximal inferior vena cava diameter, mm	14 (11–19)
Composite congestion score	1 (0–2)

Data are expressed as the median (interquartile range) or *n* (%). A, late transmitral flow velocity; E, early transmitral flow velocity; E/e’, ratio of peak mitral E wave velocity to peak early diastolic myocardial velocity at septal and lateral annuli recorded using tissue Doppler imaging.

## Data Availability

A dataset supporting the conclusions of this study has been included in the revised manuscript. Additional data are available upon request from the corresponding author.

## Abbreviations

The following abbreviations are used in this manuscript:

AUC	area under the curve
CCS	composite congestion score
HF	heart failure
IVC	inferior vena cava
LF index	liver fibrosis index
ROC	receiver operating characteristic
Vs	Vs value
V/L	Vs value/liver fibrosis index
